# Handgrip strength associates with effort-dependent lung function measures among adolescents with and without asthma

**DOI:** 10.1038/s41598-023-40320-4

**Published:** 2023-08-10

**Authors:** Laura Marie Hesselberg, Julie Nyholm Kyvsgaard, Jakob Stokholm, Hans Bisgaard, Klaus Bønnelykke, Bo Chawes

**Affiliations:** 1 COPSAC, Copenhagen Prospective Studies on Asthma in Childhood, Herlev and Gentofte Hospital, University of Copenhagen, Copenhagen, Denmark; 2 Department of Paediatrics, Slagelse Sygehus, Slagelse, Denmark; 3 Section of Microbiology and Fermentation, Department of Food Science, University of Copenhagen, Copenhagen, Denmark

**Keywords:** Physiology, Diseases, Medical research

## Abstract

Studies have shown association between handgrip strength (HGS) and FEV1, but the importance of this in relation to asthma pathophysiology and diagnostics remains unclear. We investigated the relationship between HGS and lung function metrics and its role in diagnosing asthma. We included 330 participants (mean age: 17.7 years, males: 48.7%) from the COPSAC_2000_ cohort and analyzed associations between HGS, asthma status, spirometry measures (FEV1, FVC, MMEF, FEV1/FVC), airway resistance (sRaw), methacholine reactivity (PD20) and airway inflammation (FeNO). Finally, we investigated whether HGS improved FEV1 prediction and classification of asthma status. HGS was only associated with forced flows, i.e., positive association with FEV1 and FVC for both sexes in models adjusted for age, height, and weight (*P* < 0.023). HGS improved adjusted R^2^-values for FEV1 prediction models by 2–5% (*P* < 0.009) but did not improve classification of asthma status (*P* > 0.703). In conclusion, HGS was associated with the effort-dependent measures FEV1 and FVC, but not with airway resistance, reactivity, inflammation or asthma status in our cohort of particularly healthy adolescents, which suggests that the observed associations are not asthma specific. However, HGS improved the accuracy of FEV1 estimation, which warrants further investigation to reveal the potential of HGS in asthma diagnostics.

## Introduction

Asthma is a chronic lung disease characterized by variable, reversible airflow obstruction, bronchial hyperreactivity and airway inflammation. Patients with asthma are at risk of experiencing activity limitations with reduced exercise capacity and restrictions of daily activities^1–3^. This may partly be explained by reduced physical strength, which has been suggested as a comorbidity of asthma^4,5^. This hypothesis is substantiated by studies showing that handgrip strength (HGS), which is a well-established, easy to perform and low cost measure of physical strength^6–8^, is positively associated with spirometry indices, particularly forced expiratory volume at 1 s (FEV1)^9,10^. This has been demonstrated in different populations including healthy children^11,12^, adults^9,10^ and seniors^13^ and in populations with asthma^4^.

Lung function tests constitute an important part of the diagnostic work-up for asthma with spirometry being the most widely accepted^14^, but other tests such as whole-body plethysmography, bronchodilator response, bronchial provocation tests and assessment of fractional exhaled nitric oxide (FeNO) level are also used^15^. These tests measure different aspects of asthma pathophysiology, i.e., airway obstruction, reactivity, and inflammation. Although there seems to be a well-established association between HGS and FEV1, the wider role of HGS in asthma pathophysiology and diagnostics is still unclear.

Therefore, we investigated the association between HGS, asthma status, FEV1, Forced Vital Capacity (FVC), Maximal Mid Expiratory Flow (MMEF), and the FEV1/FVC ratio, specific airway resistance (sRaw), methacholine challenge results, and FeNO level in participants from the COPSAC_2000_ mother–child cohort using data from a scheduled 18-year follow-up visit. Further, we examined whether HGS could contribute to the prediction of FEV1 and asthma diagnostics.

## Methods

### Study population

The COPSAC_2000_ cohort is a prospective single-center clinical birth cohort of 411 children born between 1998 and 2001 to mothers with a history of asthma. The recruitment procedure and baseline characteristics of the participants have been described previously^16^. The children were enrolled at 4 weeks of age and were subsequently examined at scheduled visits every 6 months until the age of 7 years and at 13 and 18 years. Data for this study was derived from a scheduled 18-year follow-up visit as this was the only visit where HGS was measured in the cohort.

### Ethics

The COPSAC_2000_ cohort study was conducted in accordance with the guiding principles of the Declaration of Helsinki and was approved by the Local Ethics Committee (KF 01-289/96), and the Danish Data Protection Agency (2015-41-3696). Both parents gave oral and written informed consent before enrolment.

### Objective assessment at age 18 years

#### Handgrip strength

Data collection was performed according to the American Society of Hand therapists guidelines^17^. A DHD-1 digital hand dynamometer ((SH1001) Saehan, Changwon, Korea) was used for measuring HGS. The dynamometer was set to position 2. The participants were seated in a chair in an upright position with the elbow in a 90° angle and the wrist in a neutral position with a maximal extension of 30°. COPSAC physicians showed these positions to the participants and gave oral instructions on how to perform the measurements. Participants were instructed to squeeze the dynamometer as hard as they could and were verbally encouraged during the procedure. Three measurements were conducted on each hand with a change of hand between each measurement. A maximal variation of 10% between attempts on each hand was accepted. Hand dominance was recorded and an average of three measurements on the dominant hand was used as a measure of HGS.

#### Lung function measurements

Spirometry: FEV1, FVC and MMEF were measured by spirometry in accordance with the ERS/ATS international guidelines using the MasterScope Pneumoscreen (Erich Jäeger, Würzburg, Germany)^14^. FEV1, FVC and FEV1/FVC-ratio are used to measure airway obstruction.

Whole-body plethysmography: sRaw, which is a measure of airway resistance, was assessed by whole-body plethysmography with the MasterScope Bodybox (Erich Jäeger, Würzburg, Germany) as previously described in detail^18^.

Methacholine challenge was done using the Vyntys APS Pro (CareFusion, 234 GmbH, Germany)^19^. The dose started at 36 μg with stepwise increases of 36 μg until 144 μg after which the dose was doubled until a final dose of 9216 μg. A three-point logistic regression model was used to estimate the cumulative methacholine dose that caused a 20% drop in FEV1 from baseline (PD20) from the dose–response curves^20^. Methacholine is a bronchoprovocation test that assess airway hyperreactivity. A low PD20 indicates hyperreactive airways.

FeNO level was measured in duplicates using the CLD 88 sp (Eco Medics, DX0256, Switzerland), in accordance with standard operating procedures^21^. FeNO is a measure of exhaled nitric oxide, which serves as a biomarker for eosinophilic airway inflammation. High FeNO levels indicate the presence of inflammation.

### Diagnosis of asthma

Asthma was solely diagnosed by the COPSAC physicians following a predefined, validated quantitative symptom-based algorithm as previously described in detail^22,23^.

### Covariates

We investigated a range of potential confounders; age, height, weight, body mass index (BMI), total muscle mass, body fat percentage, fitness (maximal oxygen consumption per kg body mass, VO_2_/kg/min), alcohol and smoking habits, screen time, self-assessed social rank, asthma, rhinitis and eczema at the 18-year visit (see Online Supplement).

### Statistics

Continuous variables exhibiting normal distribution are described as mean ± standard deviation (SD) and continuous variables not exhibiting normal distribution are described as median and interquartile range (IQR). PD20 and FeNO were log10-transformed to achieve normal distribution. Categorical variables are presented as total number and percentage. Comparisons between subgroups were done using Student’s t-test, Wilcoxon rank sum test and Chi-squared test.

The association between HGS and continuous outcomes were examined using linear regression models and log-linear regression models, whereas logistic regression was used for binary outcomes. HGS analyses are usually adjusted for sex, age, height, and weight^24^ and as these are also associated with lung function, they were chosen a priori to be included in the models. For sensitivity analyses, we further investigated the impact of a wide variety of covariates as potential confounders including covariates in the models that were associated with both HGS and outcomes.

The impact of asthma on the associations between HGS and lung function was examined by (1) adjusting the models for asthma status, (2) analyzing the data stratified by asthma status, and (3) investigating for interaction by adding cross-product to the models.

The value of HGS for improving standard prediction of FEV1 and FVC using age, height, weight and asthma was done utilizing adjusted R^2^-values and ANOVA tests.

The value of HGS for improving classification of asthma status based on FEV1 was done using receiver-operating characteristic (ROC) curves comparing areas under the curve (AUC) from logistic regression models with vs. without inclusion of HGS.

The statistical analyses were performed as complete case analyses and were done using a two-tailed test. A *P*-value ≤ 0.05 was considered significant. All statistical analyses were done using R statistical software version 64 4.0.2.

## Results

### Baseline characteristics

A total of 370 (90%) of the 411 participants in the COPSAC_2000_ cohort completed the 18-year follow-up visit. Of these 370 participants, 330 (80%) were included as they had measurements of HGS and at least one lung function outcome measure. The study population was primarily Caucasian (N = 318, 96%), median age was 17.6 years (IQR 17.4–17.9), and 161 (49%) were males. The mean HGS was 42.7 kg (SD 8.8) for males and 27.2 kg (5.4) for females with a sex difference (*P* < 0.001). Baseline characteristics and lung function results are outlined in Table 1 and Supplementary Table e1 and the correlation between HGS measures are shown in Supplementary Figure e1. The 330 included vs. 81 excluded participants had better social circumstances and a higher prevalence of allergic rhinitis (Supplementary Table e2)*.*

**Table 1:** Baseline Characteristics.

		Total, N = 330	Missing	Males, N = 161 (49%)	Missing	Females, N = 169 (51%)	Missing	*P*
*At 18 years*								
Caucasian		318 (96%)	0	153 (95%)	0	165 (98%)	0	0.333
Age (yr)		17.6 (17.4–17.9)	0	17.6 (17.4–17.9)	0	17.5 (17.4–18)	0	0.598
Height (cm)		175 (9.4)	0	182 (6.9)	0	168.5 (6.2)	0	< 0.001
Weight (kg)		67.8 (61.4–77.2)	0	72.7 (65.9–82)	0	63.9 (57.9–70.5)	0	< 0.001
Asthma diagnosis		101 (31%)	0	51 (32%)	0	50 (30%)	0	0.77
	Persistent N (%)	35 (11%)	0	19 (12%)	0	16 (9%)	0	0.61
	Intermitent N (%)	78 (24%)	0	39 (24%)	0	39 (23%)	0	0.908
Atopic dermatitis		34 (10%)	0	12 (7%)	0	22 (13%)	0	0.139
Allergic rhinitis		138 (42%)	0	71 (44%)	0	67 (40%)	0	0.479
HGS (kg)		34.7 (10.7)	1	42.7 (8.8)	1	27.2 (5.4)	0	< 0.001
FEV1 (L)		3.9 (0.8)	1	4.5 (0.6)	1	3.3 (0.5)	0	< 0.001
FVC (L)		4.4 (0.9)	1	5.1 (0.7)	1	3.7 (0.5)	0	< 0.001
MMEF (L)		4.2 (1.1)	1	4.8 (1.1)	1	3.7 (0.9)	0	< 0.001
FEV1/FVC		0.9 (0.9–42.8)	1	0.89 (0.11)	1	0.91 (0.1)	0	0.043
sRaw (kPa/s)		1.11 (0.9–1.3)	2	1.16 (1–1.3)	1	1.06 (0.9–1.2)	0	0.006
PD20 (µg)		238.6 (141.9–714.8)	79	310.9 (161.6–1073.1)	39	199.2 (128.3–464.6)	40	0.015
FeNO (ppb)		16.4 (11.2–26.7)	3	20.9 (13.1–31.6)	0	13.9 (10.1–23.3)	3	< 0.001
Key								

Data are presented as n (%) for categorical variables, mean (SD) for continuous normally distributed variables, and median (Q25:Q75) for continuous non-normally distributed variables. For categorical variables the Chi-squared test was used, for continuous normally distributed variables. Two sample t-tests were used and for normally distributed continuous variables and Wilcoxon rank sum test was used for variables not normally distributed.

FeNO = Fractional Exhaled Nitric Oxide, FEV1 = Forced Expiratory Volume 1 second, FVC = Forced Vital Capacity, HGS = Handgrip Strength, MMEF = Maximal Mid-expiratory Flow, PD20 = Provocation Dose of methacholine causing a drop of 20% in FEV1, sRaw = Specific Airway Resistance.

### Predictors of HGS

Predictors including increasing height, total muscle mass, muscle percentage, increased fitness (VO_2_/kg/min) measured by a step test and decreased body fat percentage and body fat mass were positively associated with HGS in the entire study group. Increased fitness, muscle percentage, decreased body fat percentage and body fat mass was associated with greater HGS only among males (Supplementary Table e3). Among these potential sex-specific differences in HGS predictors there were interactions between sex and body fat percentage, body fat mass, muscle mass, and fitness (*P-interactions* < 0.05).

We thereafter investigated whether the predictors of HGS were associated with any of the outcome measures. Muscle mass, muscle percentage, body fat mass, body fat percentage and fitness were associated with one or more lung function outcomes (Supplementary Table e4).

### Associations between HGS, lung function, airway hyperreactivity and inflammation

**Figure 1:**
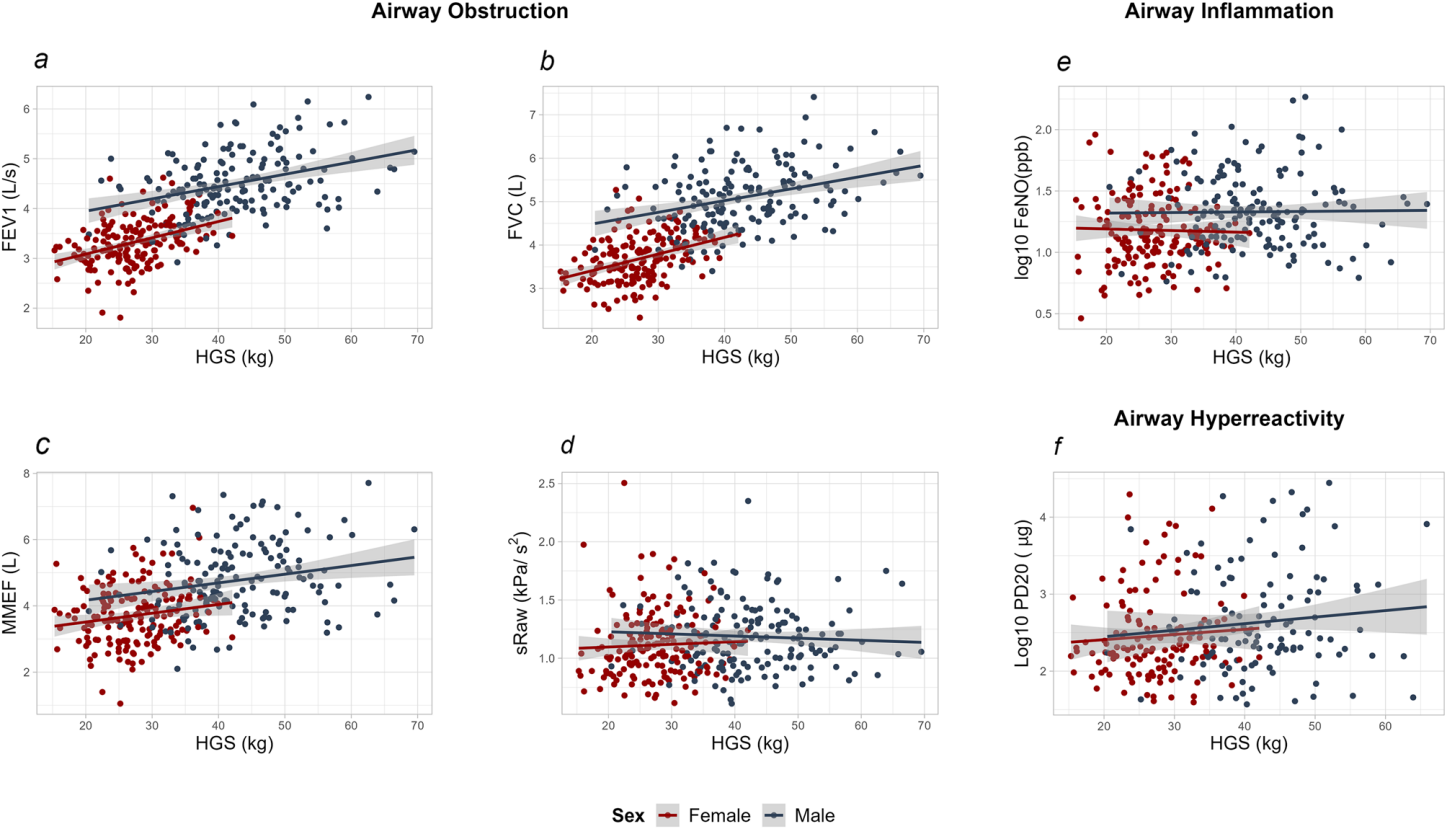
Crude Associations Between Handgrip Strength and Lung Function Tests. The figure shows scatterplot of handgrip strength spirometry measures (FEV1, FVC, MMEF), airway resistance (sRaw), airway inflammation (FeNO) and methacholine challenge test (PD20) with linear regression lines. Key: FeNO = Fractional Exhaled Nitric Oxide, FEV1 = Forced Expiratory Volume 1 second, FVC = Forced Vital Capacity, HGS = Handgrip Strength, MMEF = Maximal Mid-expiratory Flow, PD20 = Provocation Dose of methacholine causing a drop of 20% in FEV1, sRaw = Specific Airway Resistance.

In models adjusted for age, height and weight, HGS was positively associated with FEV1 (β-estimate per kg HGS, all: 0.02L, 0.009–0.022, *P* < 0.001; males: 0.01L, 0.003–0.022, *P* = 0.009; females: 0.02L, 0.009–0.031, *P* < 0.001) and FVC (all: 0.02, 0.009–0.024, *P* < 0.001, males: 0.01L, 0.002–0.023, *P* = 0.023; females: 0.02L, 0.011–0.035, *P* < 0.001), but not with MMEF, sRaw, PD20 or FeNO (Table 2). Further adjustments for use of inhaled corticosteroids, muscle mass, muscle percentage, body fat mass, body fat percentage and fitness showed similar results (Supplementary Tables e5–7). The associations between HGS, lung function, airway hyperreactivity and inflammation are visualized by scatterplots in Fig. 1.

**Table 2:** Association Between Handgrip Strength, Lung Function airway inflammation and hyperreactivity.

Outcome	All		Male		Female	
	β Estimate (95%CI)	P value	β Estimate (95%CI)	P value	β Estimate (95%CI)	P value
FEV1 (L)^†^	0.015 (0.009; 0.022)	< 0.001	0.012 (0.003; 0.022)	0.009	0.02 (0.009; 0.031)	< 0.001
FVC (L)^†^	0.017 (0.009; 0.024)	< 0.001	0.013 (0.002; 0.023)	0.023	0.023 (0.011; 0.035)	< 0.001
MMEF (L)^†^	0.015 (0; 0.029)	0.053	0.012 (−0.007; 0.032)	0.208	0.016 (−0.009; 0.041)	0.203
FEV1/FVC^†^	0 (−0.001; 0.001)	0.905	0.0 (−0.001; 0.002)	0.895	0.0 (−0.002; 0.002)	0.724
sRaw (kPa/s)^†^	−0.001 (−0.005; 0.004)	0.745	−0.002 (−0.007; 0.003)	0.473	0.002 (−0.006; 0.011)	0.574

	Exp (β Estimate) (95%CI)	P value	Exp (β Estimate) (95%CI)	P value	Exp (β Estimate) (95%CI)	P value
FeNO (ppb)^‡^	0.999 (0.989; 1.009)	0.857	1.000 (0.989; 1.014)	0.965	0.995(0.975; 1.014)	0.622
PD20 (mcg)^‡^	1.016 (0.989; 1.042)	0.234	1.023 (0.989; 1.059)	0.198	1.041 (0.951; 1.14)	0.388

Multiple linear regression was used for continuous outcomes^†^ and multiple log-linear regression was used for log transformed continuous outcomes‡ . All analyses are adjusted for age, height, and weight. Analyses performed on the overall group are further adjusted for sex.

Key: FeNO = Fractional Exhaled Nitric Oxide, FEV1 = Forced Expiratory Volume 1 second, FVC = Forced Vital Capacity, HGS = Handgrip Strength, MMEF = Maximal Mid-expiratory Flow, PD20 = Provocation Dose of methacholine causing a drop of 20% in FEV1, sRaw = Specific Airway Resistance.

### Impact of asthma on the association between HGS and lung function

There was no association between HGS and asthma status for either the entire study group or among males or females (β-estimate asthma vs. no asthma, all: −0.23 kg, −1.88 to 1.42, *P* = 0.784; males: −0.18 kg, −3.025 to 2.659, *P* = 0.899; females: −0.20 kg, −1.968 to 1.578, *P* = 0.829). Further, adding asthma as a covariate to the models did not substantially change the findings, i.e., still positive associations between HGS, FEV1 and FVC (Supplementary Table e8). Finally, there was no interaction between HGS and asthma status for FEV1 or FVC in the entire study group or among females, whereas among males there was a trend of interaction for FEV1 (*P-interaction* = 0.075) but not FVC (*P-interaction* = 0.118) (Table 3).

**Table 3:** Subgroup Analyses of Asthma versus No asthma and Interaction Analyses between Handgrip Strength and Asthma.

Outcome		All		Male		Female	
FEV1 (L)		β Estimate (95%CI)	P value	β Estimate (95%CI)	P value	β Estimate (95%CI)	P value
	Asthma	0.01 (−0.01; 0.02)	0.411	0 (−0.02; 0.02)	0.914	0.02 (0; 0.04)	0.113
	No Asthma	0.02 (0.01; 0.03)	< 0.001	0.02 (0.01; 0.03)	0.002	0.02 (0.01; 0.03)	0.003
	Interaction HGS x asthma	−0.01 (−0.02; 0.0)	0.192	−0.02 (−0.04; 0)	0.075	0.0 (−0.02; 0.02)	0.879
FVC (L)							
	Asthma	0.0 (−0.01; 0.02)	0.53	0.0 (−0.02; 0.02)	0.915	0.02 (0; 0.05)	0.079
	No Asthma	0.02 (0.0128; 0.03)	< 0.001	0.02 (0.0061; 0.03)	0.004	0.02 (0.01; 0.04)	0.001
	Interaction HGS x asthma	0.0 (−0.01; 0.01)	0.979	−0.02 (−0.04; 0)	0.118	0.0 (−0.02; 0.03)	0.91

Multivariate linear regression was used for all analyses.

Key: FEV1 = Forced Expiratory Volume 1 second, FVC = Forced Vital Capacity, HGS = Handgrip Strength.

### HGS for predicting lung function and classifying asthma status

Adding HGS to a model predicting FEV1 consisting of age, height, weight and asthma raised the adjusted R^2^ by 0.02 (*P* < 0.001) in the entire study group, 0.03 (*P* = 0.009) among males and 0.05 (*P* < 0.001) among females, suggesting that 2%, 3% and 5% of FEV1 variation is explained by HGS, respectively. The findings were similar for FVC (Table 4)*.*

**Table 4:** Prediction of FEV1 with versus without Handgrip Strength.

Outcome	All			Male			Female		
	R^2^	R^2^ (with HGS)	P value	R^2^	R^2^ (with HGS)	P value	R^2^	R^2^ (with HGS)	P value
FEV1 (L)	0.7	0.72	< 0.001	0.34	0.37	0.009	0.34	0.39	< 0.001
FVC (L)	0.71	0.73	< 0.001	0.36	0.38	< 0.001	0.36	0.41	< 0.001

Adjusted R^2^ values from multivariate linear regression models including age, height, weight and asthma status + /− HGS are used as a measure for model fit.

ANOVA-test was used to assess the differences in the model fits.

Key: ANOVA = Analyses of variance, FEV1 = Forced Expiratory Volume 1 second, HGS = Handgrip Strength.

FEV1 was associated with asthma status in the entire study group (OR per L FEV1, 0.47, 0.27–0.823,* P* = 0.009) and among females (0.38, 0.15–0.941, *P* = 0.040), but not among males (OR 0.58, 0.284–1.151, *P* = 0.126). Adding HGS to the models resulted in small changes of these estimates (all: 0.46, 0.257–0.813, *P* = 0.009; females: 0.36, 0.136–0.914, *P* = 0.034; males: 0.56, 0.267–1.126, *P* = 0.110). ROC curve analyses showed that AUC did not increase when adding HGS to the models (Table 5 and supplementary Figure e2).

**Table 5:** Classification of Asthma Status Dependent on FEV1 with versus without Handgrip Strength.

Outcome	All			Male			Female		
	AUC (95%CI)	AUC (95%CI) (with HGS)	P value	AUC (95%CI)	AUC (95%CI) (with HGS)	P value	AUC (95%CI)	AUC (95%CI) (with HGS)	P value
Asthma	0.605(0.54;0.67)	0.61(0.541;0.673)	0.856	0.576(0.54;0.67)	0.58(0.541;0.673)	0.762	0.651(0.54;0.67)	0.649(0.541;0.673)	0.846

This table displays the results of ROC analyses classifying asthma status based on FEV1. The AUC was compared for two models in each group, one where FEV1 is adjusted for age, height, and weight and one where FEV1 is adjusted for age, height, weight, and handgrip strength. The P values derives from analyses comparing the two models.

Key: AUC = Area under the curve, FEV1 = Forced Expiratory Volume 1 second, HGS = Handgrip Strength.

## Discussion

This study provides a thorough examination of the association between HGS and measures of asthma pathophysiology among 18-year-old adolescents with and without asthma. HGS was positively associated with the effort-dependent measures of FEV1 and FVC among both sexes but was not associated with any other asthma endpoints in our cohort of predominantly healthy adolescents, suggesting that the observed associations are not asthma specific. However, adding HGS to the standard spirometry prediction equation improved accuracy of FEV1 estimation, which warrants further investigation to reveal the potential of HGS in asthma diagnostics.

We found that HGS was positively associated with FEV1 and FVC in both males and females. This aligns with some previous studies^9,11,25^, whereas others found no association^26,27^ or conflicting results^28^. The ambiguous results in the literature might be due to differences in study populations, where the association seems most robust in healthy, young populations^9,11^ rather than populations consisting of aging^27^ or ill people^26,28,29^, which also fits with our findings in a largely healthy cohort of 18-year-olds.

We found a trend of association between HGS and MMEF and no association with FEV1/FVC-ratio, which is contradictory to previous studies in healthy subjects^11,30^ . MMEF is more affected by small airway obstruction than FEV1^31,32^, which may explain the discrepancy between current literature and our results as our cohort consists of adolescents who are predominantly healthy or having mild to moderate asthma.

To our knowledge, no previous studies have investigated the relationship between HGS and sRaw, PD20 or FeNO. There was no association between HGS and any of these endpoints, including asthma status, which suggests that the associations between HGS, FEV1 and FVC are not driven by underlying chronic illness of the airways, but perhaps rather an overall illustration of muscle strength. This was supported by the fact that adjusting the associations between HGS and spirometry for asthma did not affect the estimates substantially. Our subgroup analyses of participants with vs. without asthma showed the strongest association between HGS, FEV1 and FVC among participants without asthma but there was no interaction between HGS and asthma status in relation to FEV1 and FVC.

HGS was not a marker of asthma status in our study group of 18-year-old participants who were predominantly healthy or having mild to moderate asthma, which is in contrast to the sparse exiting litterature^4,5^. Our cohort has been followed prospectively since one month of age at the COPSAC clinic, and therefore we might have diagnosed more mild asthma cases and have very well-controlled asthma patients in the cohort. This might affect a possible association between asthma and HGS, although this is speculative.

A plausible mechanism behind the associations between HGS and the effort-dependent lung function measures is that HGS might be a surrogate marker of respiratory muscle strength, which might be why it is only associated with the effort-dependent spirometry measures in our study. Previous studies have shown an association between HGS and respiratory muscle strength measured as the Maximal Expiratory Pressure (MEP) and Maximal inspiratory Pressure (MIP)^33,34^. To our knowledge there a relatively few studies that have examined the relationship between MEP and MIP and spirometry indices and they found that MEP and MIP associated with increased spirometry indices^34,35^.

Even though the relationship between HGS, FEV1 and FVC seems independent of asthma status in our study, HGS may be utilized to improve estimation of lung function, which is usually based on anthropometrics including sex, age, height, ethnicity and sometimes weight^36^. The results of our study showed that adding HGS to the prediction equation for FEV1 and FVC gave more accurate predicted values with a 2–5% increase in explained variance. However, it is possible this might be due to unaccounted confounders or non-linearities. This increase in accuracy did not improve classification of asthma status in our cohort. However, our cohort consisted of predominantly healthy adolescents with few subjects with mostly mild asthma, where FEV1 was not a strong predictor of asthma status. Therefore, there is a need for larger cross-sectional studies to examine whether the use of HGS could improve diagnostics of asthma.

Interestingly, we observed several differences between males and females regarding factors associated with HGS, primarily within body composition and fitness. Body composition has previously been associated with HGS^37–40^, which aligns with our results showing association between muscle mass and HGS.

This study is strengthened by the single-center setup where all objective measurements were done by trained professionals strictly following standard operating procedures. The participants of the COPSAC_2000_ cohort have partaken in examinations repeatedly from birth till age 18 years and are therefore highly competent in performing lung function tests, which assured a high completion rate.

The examination of the association between HGS and several measures of lung function, airway resistance, reactivity, and inflammation adds to the literature as former studies have solely investigated the relationship between HGS and spirometry indices^9–12,27^. Further, the extensive exposure information made it possible to examine determinants of HGS, sex differences, and delineate potential confounders of the relationship between HGS and lung function outcomes.

One limitation of the study is the relatively low number of participants compared to some previous studies on HGS and spirometry^10,11^, which may have reduced the statistical power, mainly in the subgroup analyses of participants with vs. without asthma. However, we were still able to show a strong relationship between HGS, FEV1 and FVC in contrast to no association between HGS and measures of airway resistance, reactivity, and inflammation.

The high-risk nature of the cohort regarding asthma limits generalizability of our findings. Further, the age range was limited, and the participants were primarily of Caucasian origin. However, previous studies including participants not solely born to mothers with asthma^11^, participants of different age groups^4,10,11^, race,^9,10^ and/or residing in different geographical regions^9,10^ found results similar to ours.

## Conclusion

Handgrip strength was associated with the effort-dependent measures of FEV1 and FVC but not with airway resistance, reactivity, inflammation, or asthma status. However, adding HGS to the standard prediction equation for FEV1 improved accuracy, which warrants further investigation to reveal the potential of HGS in asthma diagnostics.

### Governance

We are aware of and comply with recognized codes of good research practice, including the Danish Code of Conduct for Research Integrity. We comply with national and international rules on the safety and rights of patients and healthy subjects, including Good Clinical Practice (GCP) as defined in the EU's Directive on Good Clinical Practice, the International Conference on Harmonisation's (ICH) good clinical practice guidelines and the Helsinki Declaration. We follow national and international rules on the processing of personal data, including the Danish Act on Processing of Personal Data and the practice of the Danish Data Inspectorate.

### Supplementary Information

Supplementary Information.

## Data Availability

The data supporting the findings of this study are available from the corresponding author upon reasonable request.

## Abbreviations

BMI: Body mass index
COPSAC_2000_: Copenhagen Prospective Studies on Asthma in Childhood_2000_
FeNO: Fractional exhaled nitric oxide
FEV1: Forced expiratory volume 1 second
FVC: Forced vital capacity
HGS: Handgrip strength
MMEF: Maximal mid-expiratory flow
PD20: Provocation dose of methacholine causing a drop of 20% in FEV1
sIgE: Specific immunoglobulin E
SPT: Skin prick test
sRaw: Specific airway resistance
