# Supplementary material for: Handgrip strength associates with effort-dependent lung function measures among adolescents with and without asthma
Source: Sci Rep. 2023 Aug 10;13:13044. doi: 10.1038/s41598-023-40320-4 (PMC10415250; doi:10.1038/s41598-023-40320-4)
Supplement: Supplementary file 1 — Supplementary Information. [file 41598_2023_40320_MOESM1_ESM.docx]

# **Supplemental Materials**

**Methods**

**Data obtained during the 18-year follow-up visit.**

Standing height in cm was measured by a Harpenden stadiometer (Holtain Ltd, Crymych, Dyfed, Wales) as the average of three measurements to the nearest two decimals.

Weight in kg was measured without clothes using a calibrated digital weighting scale.

Total muscle mass in kg and body fat in percentage was measured without clothes using a Tanita scale (health monitor version 3.3.7) body composition analyzer (Tokyo, Japan).

Fitness (ml O_2_/kg/min) was obtained by the participants performing a Danish Steptest(E1).

Information of alcohol consumption, cigarette smoking, passive smoke exposure, screen time, time spent reading and self-assessed social rank was obtained by personal interviews at the COPSAC clinic. Participants were asked to base their answers on the year leading up to the visit.

Alcohol consumption was graded according to *The Alcohol Use Disorder Identification Test* recommended by WHO(E2). The 3 first questions regarding frequency and amount of alcohol consumption were used. This gave a score from 0 to 12, with 0 being no alcohol consumption and 12 being the largest alcohol consumption.
Cigarette smoking was grouped as current smoker or no smoker. Current smoker was defined as smoking on a weekly basis.
Passive smoke exposure was grouped as exposed and not exposed. Exposure to passive smoke was defined as exposure more than 52 days per year.

Screen time was defined as the total time in hours during a week spent watching television, using a phone or using a computer in the participant’s spare time.

Self-assessed social rank was obtained by the participants rating their own social rank on a scale ranging from 1-5. This was acquired from an electronic questionnaire.
1 represented participants feeling very unpopular or low in the social hierarchy and 5 represented the participants being the most popular and socially accepted persons. For analyses, group 4 and 5 were merged as very few participants placed themselves in group 5.

Allergic rhinitis was diagnosed by COPSAC paediatricians based on interviews with the subject and their parents on history of symptoms in the subjects eighteenth year of life. It was defined as troublesome sneezing, blocked or running nose in the past year without simultaneous cold of flu(E3).

Atopic dermatitis was diagnosed by COPSAC paediatrician according to Hanifin & Rajka criteria(E4) from symptoms during subjects eighteenth year of life.

Handgrip strength data

Handgrip strength can be stated in various ways. We had 3 measures on each hand which resulted in 6 different HGS measure. Max of both hand: The maximal measurement of the 6 measurements from both hands.

Maximum HGS values of 3 measurements on the non-dominant hand.

Maximum HGS values of 3 measurements on the non-dominant hand.

Maximum HGS values of 6 measurements of both hands

The mean average of the 6 measurements from both hands.

The mean average of HGS values of 3 measurements on the non-dominant hand.

The mean average HGS values of 6 measurements of both hands

As shown in the heatmaps (figure e1A and e1B) all these different ways of defining handgrip strength are highly correlated. Therefore, we chose one definition for all our analyses, the average of the 3 measurements on the dominant hand.

**Data obtained before the 18-year follow-up**

Social circumstances such as maternal educational level, maternal age and household income was obtained by personal interviews at the COPSAC clinical research unit at a scheduled 2-year visit.

Maternal education level was divided into 3 groups based on the length of their education. Group one comprised of college level educations or lower. Group two entailed medium length educations and the third group was university educations or higher.
Household income was defined as the yearly income in DDK in the household of the participant’s primary home. It was grouped as follows: <400.000, 400.000- 600.000, 600.000-800.000, 800.000-1000.000 and > 1000.000.
Social circumstances scale is the result of a principal component analysis of maternal age, maternal education and household income at age 2years.

**Statistics**

Univariable regression analyses between HGS and lung function and allergy outcomes

The association between HGS and FEV1, FVC, MMEF, FEV1/FVC, sRaw, FeNO and PD20 were initially examined using linear regression models. Linearity of the associations were visually assessed by plotting model residuals against the predictor variables and fitting a LOESS curve. Assumptions of homoscedasticity, independence and normality were met. Logistic regression model was used to examine HGS association with allergic sensitization and skin prick test. For the multivariate regression analyses ordinary least squares linear regression was used for FEV1, FVC, FEV1/FVC, NNEF, sRAW and FeNO, weighted least squares regression was used for PD20 and multivariate logistic regression for allergic sensitization and skin prick test.

Potential confounders

Selection of potential confounders was done by performing univariate analyses of association with HGS in the male and female subgroups. Analyses in the *All* group were adjusted for sex. Linear regression analysis was used for continuous and factorial variables and Student’s t-test for categorial. If variables were significantly associated with HGS, they were then examined for association with outcomes of lung functions measures firstly as crude analyses and if they showed association then by analyses adjusted for age, height and weight which are the most common covariates of lung function measure. Covariates exhibiting association with both HGS, and measures of lung function were selected as potential confounders.

Multivariable regression with selected confounders

The selection of potential confounder yielded following models. Height, weight and age were all kept as part of the models as these are known confounders. ***(Table e5 and e6)***. For the overall group four measures of body composition which were all highly correlated were selected potential confounders. Therefor a model with all potential confounders and four distinct models, with only one of these body composition measures.

**Results**

Univariable regression analyses between HGS and lung function and allergy outcomes

In both the overall group, males and females, HGS was significantly associated with FEV1 (per kg HGS, all: 0.05L, CI 95%: 0.05-0.06, *P*<0.001, males: 0.03L, 0.01-0.04, *P*<0.001; females: 0.03L, 0.02-0.05, *P*<0.001), FVC (all: 0.06L, CI 95%: 0.06-0.07, *P*<0.001,males: 0.03L, 0.02-0.04, *P*<0.001; females: 0.04L, 0.03-0.05, *P*<0.001) and MMEF (all: 0.05L, CI 95%: 0.04-0.06, *P*<0.001,males: 0.03L/s, 0.01-0.05, *P*=0.007; females: 0.03L/s, 0.001-0.051, *P*=0.032). HGS was significantly associated with FeNO (GMR: 1.04, CI 95%:1.02-1.06, *P*=0.001) and with PD20(1.02, CI 95%:1.01-1.04, *P*=0.009) for the overall group only. There were no significant associations between HGS and FEV1/FVC-ratio, sRaw, SPT or sIgE (***Table e9***)*.*

Multivariable regressions with selected confounders

Adjusting the associations between HGS and FEV1, FVR or MMEF for potential confounders found in the initial analyses did not change the results drastically from association only adjusted for age, height and weight. ***(Table 2, e5 and e6)***

**RFERENCES**

E1. Aadahl M, Zacho M, Linneberg A, Thuesen BH, Jørgensen T. Comparison of the Danish step test and the watt-max test for estimation of maximal oxygen uptake: the Health2008 study. Eur J Prev Cardiol. 2013 Dec;20(6):1088–94.

E2. Saunders JB, Aasland OG, Babor TF, de la Fuente JR, Grant M. Development of the Alcohol Use Disorders Identification Test (AUDIT): WHO Collaborative Project on Early Detection of Persons with Harmful Alcohol Consumption--II. Addict Abingdon Engl. 1993 Jun;88(6):791–804.

E3. Chawes BLK, Kreiner-Møller E, Bisgaard H. Objective assessments of allergic and nonallergic rhinitis in young children. Allergy. 2009 Oct;64(10):1547–53.

E4. Hanifin JM, Rajka G. Diagnostic Features of Atopic Dermatitis. Acta Dermatover Stockh. 1980;92:44–7.

E5. Maršál K, Persson P-H, Larsen T, Lilja H, Selbing A, Sultan B. Intrauterine growth curves based on ultrasonically estimated foetal weights. Acta Paediatr. 1996 Jul;85(7):843–8.

|  | Table e1: Baseline Characteristics  Data are presented as n (%) for categorical variables, mean (SD) for continuous normally distributed variables, and median (Q25:Q75) for continuous non-normally distributed variables. For categorical variables the Chi-squared test was used, for continuous normally distributed variables. Student’s t-test was used and for continuous variables that are not normally distributed a Wilcoxon rank sum test was used. | | | | | | | | |  |
| --- | --- | --- | --- | --- | --- | --- | --- | --- | --- | --- |
|  |  |  | **Total,**  **N=330** | **Missing** | **Males,**  **N=161 (49%)** | **Missing** | **Females,**  **N=169 (51%)** | **Missing** | ***P value*** |  |
|  | **At 18 years** | | | | | | | | |  |
|  | BMI (kg/m^2^) |  | 22.3 (20–24.9) | 0 | 22.1 (19.9–24.4) | 0 | 22.6 (20–25) | 0 | 0.27 |  |
|  | Body Fat % |  | 23.6 (16.9–29.1) | 15 | 22.1 (19.9–24.4) | 0 | 22.6 (20–25) | 0 | <0.001 |  |
|  | Body fat mass (kg) |  | 15.4 (11.3–20.5) | 15 | 12.2 (9.2–16) | 7 | 18 (14.2–22) | 8 | <0.001 |  |
|  | Muscle % |  | 72.2 (7.8) | 15 | 77.6 (5.8) | 7 | 67 (5.8) | 8 | <0.001 |  |
|  | Total muscle mass (kg) |  | 50.5 (9.8) | 15 | 58 (7.6) | 7 | 43.4 (5.2) | 8 | <0.001 |  |
|  | Alcohol consumption  (Numeric scale 1-10) |  | 6.1 (2.3) | 21 | 6.4 (2.4) | 8 | 5.8 (2.3) | 13 | 0.022 |  |
|  | Current smoking |  | 56 (17%) | 0 | 30 (19%) | 0 | 26 (15%) | 0 | 0.523 |  |
|  | Exposure to passive  smoke during 18th year of life |  | 110 (33%) | 0 | 54 (34%) | 0 | 56 (33%) | 0 | 1.000 |  |
|  | Self-assessed social rank |  |  | 2 |  | 1 |  | 2 | 0.031 |  |
|  |  | Low | 20 (6%) | 2 | 6 (4%) | 1 | 14 (8%) | 1 |  |  |
|  |  | Medium low | 121 (37%) | 2 | 52 (33%) | 1 | 69 (41%) | 1 |  |  |
|  |  | Medium high | 150 (46%) | 2 | 78 (49%) | 1 | 72 (43%) | 1 |  |  |
|  |  | High | 37 (11%) | 2 | 24 (15%) | 1 | 13 (8%) | 1 |  |  |
|  | Screen time (hours per week) |  | 41 (33.5–49) | 0 | 44 (35–44.5) | 0 | 38 (31–44.5) | 0 | <0.001 |  |
|  | Fitness (mlO_2_/kg/min) |  | 35 (29–42.8) | 32 | 36.5 (30.8–46) | 13 | 33 (28–39.8) | 19 | 0.003 |  |
|  | Skin prick test,  inhaled allergens (y/n) |  | 159 (49%) | 7 | 92 (58%) | 2 | 67 (41%) | 5 | 0.003 |  |
|  | Serum IgE,  inhaled allergens (y/n) |  | 0 (53%) | 9 | 97 (62%) | 5 | 72 (44%) | 4 | 0.001 |  |
|  | Social circumstances scale, PCA |  | 0.1 (1) | 13 | -0.1 (1) | 9 | 0.2 (1) | 4 | 0.025 |  |
|  | **Key**  **BMI** = Body mass index, **sIgE** = Specific Immunoglobulin E, **SPT** = Skin Prick Test, **y/n** = Yes or NO | | | | | | | | |  |

| Table e2. Comparison of included vs. excluded cohort participants  Data are presented as n (%) for categorical variables, mean (SD) for continuous normally distributed variables, and median (Q25:Q75) for continuous non-normally distributed variables. For categorical variables the Chi-squared test was used, for continuous normally distributed variables Two sample t-test was used and for continuous variables that are not normally distributed a Wilcoxon rank sum test was used. | | | | | | | | |
| --- | --- | --- | --- | --- | --- | --- | --- | --- |
|  | | | | | | | | |
| Characteristics |  | **Total N = 411** | **Missing** | **Included N = 330** | **Missing** | **Excluded N = 81** | **Missing** | ***P value*** |
| Male |  | 203 (49%) | 0 (0%) | 161 (49%) | 0 (0%) | 39 (48%) | 0 (0%) | 0.711 |
| Caucasian |  | 397 (96.59%) | 0 (0%) | 318 (96.36%) | 0 (0%) | 79 (97.53%) | 0 (0%) | 0.859 |
| Height (cm) |  | 174.9 (9.4) | 45 (10.95%) | 175 (9.4) | 0 (0%) | 173.9 (9.5) | 45 (55.55%) | 0.5 |
| Weight (kg) |  | 67.8 (61.4–77.4) | 45 (10.95%) | 67.85 (61.4–77.2) | 0 (0%) | 66.65 (60.3–78.3) | 45 (55.55%) | 0.81 |
| BMI (kg/m^2^) |  | 22.3 (20–24.9) | 45 (10.95%) | 22.3 (20–24.9) | 0 (0%) | 22 (20.7–24.1) | 45 (55.55%) | 0.919 |
| Body Fat % |  | 23.3 (16.7–29.2) | 62 (15.1%) | 23.6 (16.9–29.1) | 15 (4.5%) | 21.4 (16.6–30) | 47 (58%) | 0.706 |
| Bodyfat mass (kg) |  | 15.1 (11.2–20.3) | 62 (15.1%) | 15.4 (11.3–20.5) | 15 (4.5%) | 14.3 (11–19.7) | 47 (58%) | 0.626 |
| Muscle % |  | 72.4 (66.7–79.1) | 62 (15.1%) | 72.4 (67–79) | 15 (4.5%) | 74.6 (66.4–79.3) | 47 (58%) | 0.728 |
| Total muscle mass (kg) |  | 50.6 (9.8) | 62 (15.1%) | 50.5 (9.8) | 15 (4.5%) | 51.3 (10) | 47 (58%) | 0.659 |
| Alcohol intake numeric (1-10) |  | 6.1 (2.3) | 66 (16.1%) | 6.1 (2.3) | 21 (6.4%) | 6.1 (2.3) | 45 (55.6%) | 0.938 |
| Smoking |  | 65 (17.57%) | 41 (10%) | 56 (16.97%) | 0 (0%) | 9 (22.5%) | 41 (51%) | 0.517 |
| Exposure to passive smoke during 18th year of life |  | 126 (34.05%) | 41 (10%) | 110 (33.33%) | 0 (0%) | 16 (40%) | 41 (51%) | 0.507 |
| Self assessed socialrank |  |  | 44 (10.7%) |  | 2 (0.6%) |  | 44 (10.7%) | 0.178 |
|  | Low | 23 (6.27%) | 44 (11%) | 20 (6.1%) | 2 (1%) | 3 (7.69%) | 42 (52%) |  |
|  | Medium low | 142 (38.69%) | 44 (11%) | 121 (36.89%) | 2 (1%) | 21 (53.85%) | 42 (52%) |  |
|  | Medium high | 162 (44.14%) | 44 (11%) | 150 (45.73%) | 2 (1%) | 12 (30.77%) | 42 (52%) |  |
|  | High | 40 (10.9%) | 44 (11%) | 37 (11.28%) | 2 (1%) | 3 (7.69%) | 42 (52%) |  |
| screentime hours per week) |  | 41 (33.5–49) | 46 (11.2%) | 41 (33.5–50) | 0 (0%) | 41 (34–50) | 46 (56.8%) | 0.713 |
| Fitness (mlO_2_/kg/min) |  | 35 (29–42) | 92 (22.4%) | 35 (29–42.8) | 32 (9.7%) | 32 (26–40) | 60 (74.1%) | 0.243 |
| Current asthma diagnosis |  | 113 (30.46%) | 40 (10%) | 101 (30.61%) | 0 (0%) | 12 (29.27%) | 40 (49%) | 1.00 |
| Atopic dermatitis |  | 37 (9%) | 0 (0%) | 34 (10.3%) | 0 (0%) | 3 (3.7%) | 0 (0%) | 0.1 |
| Allergic rhinitis |  | 155 (37.71%) | 0 (0%) | 138 (41.82%) | 0 (0%) | 17 (20.99%) | 0 (0%) | 0.001* |
| FEV1 (L/1sec) |  | 3.9 (0.8) | 47 (11.4%) | 3.9 (0.8) | 1 (0.3%) | 3.8 (0.8) | 46 (56.8%) | 0.364 |
| FVC (L) |  | 4.3 (0.9) | 47 (11.4%) | 4.4 (0.9) | 1 (0.3%) | 4.2 (0.8) | 46 (56.8%) | 0.168 |
| MMEF (L/s) |  | 4.2 (1.1) | 47 (11.4%) | 4.2 (1.1) | 1 (0.3%) | 4.3 (1.2) | 46 (56.8%) | 0.693 |
| FEV1/FVC |  | 0.91 (0.9–42) | 47 (11.4%) | 0.9 (0.1) | 1 (0.9–42.8) | 0.93 (0.09) | 46 (0.9:40) | 0.125 |
| sRAW (kPa/s^2^) |  | 1.1 (0.9–1.3) | 48 (11.7%) | 1.11 (0.9–1.3) | 2 (0.6%) | 1.05 (0.9–1.3) | 46 (56.8%) | 0.236 |
| PD20 (µg) |  | 247.1 (142.5–702.4) | 139 (33.8%) | 238.6 (141.9–714.8) | 79 (23.9%) | 277.6 (164.8–498.8) | 60 (74.1%) | 0.638 |
| FeNO (ppb) |  | 16.6 (11.2–27) | 48 (11.7%) | 16.4 (11.2–26.7) | 3 (0.9%) | 16.8 (10.9–30) | 45 (55.6%) | 0.84 |
| Sensitisation by  skin prick test inhalation allergens (y/n) |  | 175 (48.88%) | 53 (13%) | 159 (49.23%) | 7 (2%) | 16 (45.71%) | 46 (57%) | 0.828 |
| Sensitisation by  sIgE inhalation allergens (y/n) |  | 187 (52.68%) | 56 (14%) | 169 (52.65%) | 9 (3%) | 18 (52.94%) | 47 (58%) | 1.00 |
| Social circumstances scale (PCA-score) |  | 0 (1) | 29 (7.1%) | 0.1 (1) | 13 (3.9%) | -0.3 (1) | 16 (19.8%) | 0.027* |
| * Significant *P*-value ≤ 0.05  Key  BMI = Body mass index, FeNO = Fractional Exhaled Nitric Oxide, FEV1 = Forced Expiratory Volume 1 second, FVC = Forced Vital Capacity, HGS = Handgrip Strength, MMEF = Maximal Mid-expiratory Flow, PD20 = Provocation Dose of methacholine causing a drop of 20% in FEV1, sIgE = Specific Immunoglobulin E, SPT = Skin Prick Test, sRaw = Specific Airway Resistance, y/n = Yes or No | | | | | | | | |

| Table e3: Predictors of HGS  Linear regressions were used for continuous and factorial predictors, Student’s t-test was used for binary predictors.  Analyses in *All* group was adjusted for sex. | | | | | | | | | | |
| --- | --- | --- | --- | --- | --- | --- | --- | --- | --- | --- |
|  | **All** |  |  | **Male** |  |  | **Female** |  |  | **Interaction with sex** |
| Predictors | **Estimate (95%CI)** | ***P* value** |  | **Estimate (95%CI)** | ***P* value** |  | **Estimate (95%CI)** | ***P* value** |  | ***P* value** |
| Age(yr) | 2.03(0.58-3.48) | 0.006* |  | 2.28(-0.22-4.78) | 0.073 |  | 1.78(0.25-3.3) | 0.023* |  | 0.731 |
| Height(cm) | 0.37(0.19-0.42) | <0.001* |  | 0.385(0.193-0.578) | <0.001* |  | 0.217(0.087-0.347) | 0.001* |  | 0.158 |
| Weight(kg) | 0.05(-0-01-0.11) | 0.118 |  | 0.034(-0.062-0.131) | 0.484 |  | 0.068(-0.002-0.137) | 0.057 |  | 0.594 |
| BMI (kg/m^2^) | -0.04(-0.23-0.16) | 0.698 |  | -0.147(-0.495-0.201) | 0.406 |  | 0.055(-0.143-0.253) | 0.586 |  | 0.31 |
| Muscle mass (kg) | 0.33(0.22-0.46) | <0.001* |  | 0.311(0.132-0.49) | 0.001* |  | 0.392(0.251-0.532) | <0.001* |  | 0.554 |
| Muscle % | 0.28(0.14-0.41) | <0.001* |  | 0.458(0.224-0.692) | <0.001* |  | 0.1(-0.036-0.236) | 0.15 |  | 0.011* |
| Bodyfat mass (kg) | -0.11(-0.22- -0.01) | 0.030* |  | -0.223(-0.395- -0.05) | 0.012* |  | 0.004(-0.099-0.106) | 0.942 |  | 0.032* |
| Bodyfat % | -0.26(-0.39- -0.13) | <0.001* |  | -0.425(-0.645- -0.205) | <0.001* |  | -0.089(-0.219-0.041) | 0.179 |  | 0.012* |
| Alcohol intake numeric (1-10) | 0.18(-0.17-9.54) | 0.306 |  | 0.108(-0.482-0.699) | 0.718 |  | 0.265(-0.123-0.654) | 0.179 |  | 0.662 |
| Current smoking | -1.70(-3.81-0.40) | 0.112 |  | -1.708(-1.83-5.247 | 0.342 |  | -1.698(-0.582-3.979 | 0.143 |  | 0.996 |
| Passive smoke | -0.05(-1.72-1.63) | 0.953 |  | -0.53(-3.457-2.398) | 0.721 |  | 0.604(-1.152-2.361) | 0.498 |  | 0.508 |
| Self-assessed socialrank |  |  |  |  |  |  |  |  |  |  |
| Level 2 | -1.66(-5.10-1.78) | 0.343 |  | -1.09(-8.55-6.38) | 0.774 |  | -2.02(-5.17-1.29) | 0.207 |  | 0.805 |
| Level 3 | -1.40(-4.81-2.01) | 0.419 |  | -1.72(-9.05-5.60) | 0.643 |  | -0.92(-4.06-2.22) | 0.563 |  | 0.830 |
| Level 4 | 2.32(-1.68-6.31) | 0.225 |  | 3.50(-4.39-11.39) | 0.382 |  | 0.41(-3.73-4.55) | 0.884 |  | 0.475 |
| Screentime(hours per week) | -0.05(-0.1-0.01) | 0.086 |  | -0.076(-0.159-0.008) | 0.075 |  | -0.001(-0.068-0.066) | 0.98 |  | 0.191 |
| Fitness (mlO_2_ /kg/min) | 0.18(0.09-0.27) | <0.001 |  | 0.263(0.118-0.409) | <0.001* |  | 0.077(-0.026-0.179) | 0.141 |  | 0.044* |
| Socialcircumstances scale(pca-score) | -0.48(-1.3- -0.34) | 0.247 |  | -0.712(-2.191-0.767) | 0.343 |  | -0.288(-1.121-0.545) | 0.496 |  | 0.611 |
| Asthma | -0.39(-2.10-1.32) | 0.656 |  | 0.77(-2.199-3.74) | 0.609 |  | 0.014(-1.8-1.828) | 0.988 |  | 0.666 |
| Atopic dermatitis | 0.44(-2.17-3.06) | 0.741 |  | 1.284(-3.97-6.539) | 0.63 |  | -1.44(-3.89-1.012) | 0.248 |  | 0.324 |
| Allergic rhinitis | 1.29(-0.311-2.89) | 0.114 |  | 1.214(-1.567-3.995) | 0.39 |  | 1.365(-0.315-3.044) | 0.111 |  | 0.926 |
| * Significant *P*-value ≤ 0.05  Key  BMI = Body Mass Index, FeNO = Fractional Exhaled Nitric Oxide, FEV1 = Forced Expiratory Volume 1 second, FVC = Forced Vital Capacity, HGS = Handgrip Strength, IQR = Interquartile Range, MMEF = Maximal Mid-expiratory Flow, N = Number, PD20 = Provocation Dose of methacholine causing a drop of 20% in FEV1, sIgE = Specific Immunoglobulin E, SD = Standard Deviation, SPT = Skin Prick Test, sRaw = Specific Airway Resistance | | | | | | | | | | |

| Table e4: Association between potential confounder and lung function measurements  For continuous predictor variables linear and multiple linear regression was used. For binary predictors logistic and multiple logistic regression was used. | | | | | | | | | | | | | | |
| --- | --- | --- | --- | --- | --- | --- | --- | --- | --- | --- | --- | --- | --- | --- |
|  | **All** |  |  |  |  | **Male** |  |  |  |  | **Female** |  |  |  |
|  | **Estimate (CI95%)** | ***P*** | **Estimate (CI95%)**^†^ | ***P*** ^†^ |  | **Estimate (CI95%)** | ***P*** | **Estimate (CI95%)** ^†^ | ***P*** ^†^ |  | **Estimate (CI95%)** | ***P*** | **Estimate (CI95%)** ^†^ | ***P*** ^†^ |
| *FEV1* |  |  |  |  |  |  |  |  |  |  |  |  |  |  |
| Muscle mass | 0.059 (0.053-0.065) | <0.001* | 0.032 (0.012-0.052) | 0.002* |  | 0.028 (0.016-0.04) | <0.001* | 0.023 (-0.008-0.054) | 0.5 |  | 0.04 (0.028-0.053) | <0.001* | 0.05 (0.023-0.077) | <0.001* |
| Muscle % | 0.056 (0.047-0.066) | <0.001* | 0.021 (0.004-0.037) | 0.016* |  | 0.011 (-0.005-0.028) | 0.183 | 0.012 (-0.016-0.04) | 0.382 |  |  |  |  |  |
| Bodyfat mass (kg) | -0.022 (-0.032--0.012) | <0.001* | -0.029  (-0.048--0.01) | 0.003* |  | -0.001 (-0.013-0.011) | 0.843 | 0.003 (-0.006-0.013) | 0.465 |  |  |  |  |  |
| Bodyfat % | -0.052 (-0.061--0.043) | <0.001* | -0.018  (-0.034-0.002) | 0.030* |  | -0.01 (-0.025-0.006) | 0.219 | -0.012 (-0.038-0.015) | 0.393 |  |  |  |  |  |
| Fitness (ml O_2_/kg/min) | 0.013 (0.003-0.023) | 0.009* | 0.003 (-0.003-0.01) | 0.316 |  | 0.004 (-0.006-0.015) | 0.413 | 0.007 (-0.003-0.016) | 0.151 |  |  |  |  |  |
| *FVC* |  |  |  |  |  |  |  |  |  |  |  |  |  |  |
| Musclemass | 0.073 (0.067-0.08) | <0.001* | 0.051 (0.028-0.073) | <0.001* |  | 0.04 (0.026-0.053) | <0.001* | 0.048 (0.014-0.083) | 0.007* |  | 0.057(0.044-0.07) | <0.001* | 0.064(0.035-0.094) | <0.001* |
| Muscle % | 0.063 (0.052-0.074) | <0.001* | 0.031 (0.012-0.049) | 0.002* |  | 0.01 (-0.009-0.029) | 0.304 | 0.03 (-0.002-0.062) | 0.065 |  |  |  |  |  |
| Bodyfat mass(kg) | -0.021 (-0.034--0.009) | <0.001* | -0.045 (-0.066--0.023) | <0.001* |  | 0.002 (-0.012-0.016) | 0.785 | 0.01 (-0.001-0.021) | 0.062 |  |  |  |  |  |
| Bodyfat % | -0.059 (-0.069--0.048) | <0.001* | -0.026 (-0.044--0.008) | 0.005* |  | -0.008 (-0.026-0.01) | 0.39 | -0.026 (-0.056-0.004) | 0.094 |  |  |  |  |  |
| Fitness (ml O_2_/kg/min) | 0.017 (0.005-0.028) | 0.005* | 0.009 (0.002-0.017) | 0.015* |  | 0.008 (-0.004-0.02) | 0.19 | 0.014 (0.004-0.024) | 0.008* |  |  |  |  |  |
| *MMEF* |  |  |  |  |  |  |  |  |  |  |  |  |  |  |
| Musclemass | 0.052 (0.04-0.063) | <0.001* | 0.032 (-0.011-0.075) | 0.146 |  | 0.024 (0.001-0.047) | 0.038* | -0.005 (-0.07-0.06) | 0.876 |  | 0.026 (0-0.052) | 0.049* | 0.075 (0.015-0.136) | 0.015* |
| Muscle % | 0.054 (0.04-0.069) | <0.001* | 0.027 (-0.009-0.062) | 0.141 |  | 0.011 (-0.019-0.041) | 0.483 | 0.011 (-0.019-0.041) | 0.483 |  |  |  |  |  |
| Bodyfat mass(kg) | -0.023 (-0.038--0.009) | 0.002* | -0.027 (-0.068-0.014) | 0.192 |  | -0.001 (-0.022-0.021) | 0.956 | -0.006 (-0.023-0.012) | 0.521 |  |  |  |  |  |
| Bodyfat % | -0.05 (-0.064--0.036) | <0.001* | -0.022 (-0.056-0.012) | 0.211 |  | -0.01 (-0.038-0.019) | 0.505 | 0.003 (-0.052-0.058) | 0.912 |  |  |  |  |  |
| Fitness (ml O_2_/kg/min) | 0.005 (-0.009-0.019) | 0.470 | -0.008 (-0.022-0.006) | 0.263 |  | -0.004 (-0.023-0.015) | 0.684 | -0.004 (-0.023-0.015) | 0.683 |  |  |  |  |  |
| * Significant *P*-value ≤ 0.05  ^†^Analyses were adjusted for age, height and weight and further adjusted for sex in the *all* group.  Key  BMI = Body Mass Index, FEV1 = Forced Expiratory Volume 1 second, FVC = Forced Vital Capacity, HGS = Handgrip Strength, MMEF = Maximal Mid-expiratory Flow | | | | | | | | | | | | | | |

| Table e5: Association between HGS and lung function measurements adjusted for potential confounders – all  All analyses were performed as multiple linear regression. | | | |
| --- | --- | --- | --- |
|  | **β estimate (95%CI)** | ***P* value** | **Adjusted R^2^** |
| FEV1 |  |  |  |
| *HGS* adjusted by sex, age, height, weight, muscle mass, muscle %,  fat mass and fat % | 0.011 (0.004-0.019) | 0.004* | 0.709 |
| *HGS* adjusted by sex, age, height, weight and muscle % | 0.012 (0.004-0.019) | 0.002* | 0.709 |
| *HGS* adjusted by sex, age, height, weight and muscle % | 0.013(0.006-0.02) | <0.001* | 0.708 |
| *HGS* adjusted by sex, age, height, weight and fat mass | 0.012 (0.005-0.02) | 0.002* | 0.708 |
| *HGS* adjusted by age, height, weight and fat % | 0.013 (0.006-0.021) | <0.001* | 0.707 |
| FVC |  |  |  |
| *HGS* adjusted by sex, age, height, weight, muscle mass, muscle %,  fat mass, fat % and fitness, | 0.009 (0.0-0.018) | 0.054 | 0.728 |
| *HGS* adjusted by sex, age, height, weight, muscle % and fitness | 0.009 (0.0-0.018) | 0.06 | 0.73 |
| *HGS* adjusted by sex, age, height, weight, muscle % and fitness | 0.011 (0.002-0.02) | 0.021* | 0.726 |
| *HGS* adjusted by sex, age, height, weight, fat mass and fitness | 0.009 (0.0-0.018) | 0.05 | 0.728 |
| *HGS* adjusted by sex, age, height, weight, fat % and fitness | 0.011 (0.002-0.02) | 0.017* | 0.724 |
| * Significant *P*-value ≤ 0.05  Key  FEV1 = Forced Expiratory Volume 1 second, FVC = Forced Vital Capacity, HGS = Handgrip Strength, MMEF = Maximal Mid-expiratory Flow | | | |

| **Table e6: Association between HGS and lung function measurements adjusted for potential confounders** **– Male and Female**  All analyses were performed as multiple linear regression. | | | | | | | | |  |
| --- | --- | --- | --- | --- | --- | --- | --- | --- | --- |
| **Male** |  |  |  |  | **Female** |  |  |  |  |
|  | **β estimate (95%CI)** | ***P*** | **Adjusted R^2^** |  |  | **β estimate (95%CI)** | ***P*** | **Adjusted R^2^** |  |
|  |  |  |  |  | **FEV1** |  |  |  |  |
|  |  |  |  |  | Age, height, weight and  muscle mass | 0.013 (0.002; 0.025) | 0.027* | 0.393 |  |
| **FVC** |  |  |  |  | **FVC** |  |  |  |  |
| ***HGS*** adjusted by  age, height, weight, fitness  and  muscle mass | 0.004 (-0.009; 0.016) | 0.586 | 0.382 |  | Age, height, weight and  muscle mass | 0.015 (0.002; 0.028) | 0.024* | 0.442 |  |
|  |  |  |  |  | **MMEF** |  |  |  |  |
|  |  |  |  |  | Age, height, weight and  muscle mass | 0.003 (-0.024; 0.03) | 0.829 | 0.111 |  |
| * Significant *P*-value ≤ 0.05  **Key**  **FEV1** = Forced Expiratory Volume 1 second, **FVC** = Forced Vital Capacity, **HGS** = Handgrip Strength, **MMEF** = Maximal Mid-expiratory Flow | | | | | | | | |  |

| Table e9. Association between Handgrip Strength and FeNO adjusted for ICS use. Multiple linear regression was used for continuous outcomes^†^, multiple log-linear regression was used for log transformed continuous outcomes‡ and multiple logistic regression was for binary outcomes^§^. All analyses are adjusted for age, height, weight and ICS use. Analyses performed on the overall group are further adjusted for sex. | | | | | | | | |
| --- | --- | --- | --- | --- | --- | --- | --- | --- |
|  | **All** |  |  | **Male** |  |  | **Female** |  |
| Outcome | **Exp (β Estimate) (95%CI)** | **P value** |  | **Exp (β Estimate) (95%CI)** | **P value** |  | **Exp (β Estimate) (95%CI)** | **P value** |
| *FeNO (ppb)*^‡^ | -0.001(-0.006:0.004) | 0.642 |  | 0.001(-0.006:0.007) | 0.856 |  | -0.006(-0.015:0.003) | 0.187 |
| Key  FeNO = Fractional Exhaled Nitric Oxide, HGS = Handgrip Strength, ICS = Inhaled Corticoid Steroids | | | | | | | | |
| Table e9. Association between Handgrip Strength and FeNO adjusted for ICS use. Multiple linear regression was used for continuous outcomes^†^, multiple log-linear regression was used for log transformed continuous outcomes‡ and multiple logistic regression was for binary outcomes^§^. All analyses are adjusted for age, height, weight and ICS use. Analyses performed on the overall group are further adjusted for sex. | | | | | | | | |
|  | **All** |  |  | **Male** |  |  | **Female** |  |
| Outcome | **Exp (β Estimate) (95%CI)** | **P value** |  | **Exp (β Estimate) (95%CI)** | **P value** |  | **Exp (β Estimate) (95%CI)** | **P value** |
| *FeNO (ppb)*^‡^ | -0.001(-0.006:0.004) | 0.642 |  | 0.001(-0.006:0.007) | 0.856 |  | -0.006(-0.015:0.003) | 0.187 |
| Key  FeNO = Fractional Exhaled Nitric Oxide, HGS = Handgrip Strength, ICS = Inhaled Corticoid Steroids | | | | | | | | |
| Table e7. Association between Handgrip Strength and FeNO adjusted for ICS use. Multiple linear regression was used for continuous outcomes^†^, multiple log-linear regression was used for log transformed continuous outcomes‡ and multiple logistic regression was for binary outcomes^§^. All analyses are adjusted for age, height, weight and ICS use. Analyses performed on the overall group are further adjusted for sex. | | | | | | | | |
|  | **All** |  |  | **Male** |  |  | **Female** |  |
| Outcome | **Exp (β Estimate) (95%CI)** | **P value** |  | **Exp (β Estimate) (95%CI)** | **P value** |  | **Exp (β Estimate) (95%CI)** | **P value** |
| *FeNO (ppb)*^‡^ | -0.001(-0.006:0.004) | 0.642 |  | 0.001(-0.006:0.007) | 0.856 |  | -0.006(-0.015:0.003) | 0.187 |
| * Significant *P*-value ≤ 0.05  Key  FeNO = Fractional Exhaled Nitric Oxide, HGS = Handgrip Strength, ICS = Inhaled Corticoid Steroids | | | | | | | | |

| Table e8. Association Between Handgrip Strength, Lung Function airway inflammation and hyperreactivity adjusted for asthma. Multiple linear regression was used for continuous outcomes^†^, multiple log-linear regression was used for log transformed continuous outcomes‡ and multiple logistic regression was for binary outcomes^§^. All analyses are adjusted for age, height, weight and persistent asthma. Analyses performed on the overall group are further adjusted for sex. | | | | | | | | |
| --- | --- | --- | --- | --- | --- | --- | --- | --- |
|  | **All** |  |  | **Male** |  |  | **Female** |  |
| Outcome | **β Estimate (95%CI)** | **P value** |  | **β Estimate (95%CI)** | **P value** |  | **β Estimate (95%CI)** | **P value** |
| *FEV1 (L)*^†^ | 0.015(0.009:0.022) | <0.001* |  | 0.012(0.003:0.022) | 0.01* |  | 0.02(0.01:0.031) | <0.001* |
| *FVC (L)*^†^ | 0.017(0.009:0.024) | <0.001* |  | 0.017(0.009:0.024) | <0.001* |  | 0.023(0.011:0.035) | <0.001* |
| *MMEF (L)*^†^ | 0.014(-0.001:0.028) | 0.059 |  | 0.012(-0.008:0.031) | 0.239 |  | 0.016(-0.008:0.04) | 0.189 |
| *FEV1/FVC*^†^ | 0(-0.001:0.001) | 0.826 |  | 0(-0.001:0.001) | 0.979 |  | 0(-0.002:0.002) | 0.73 |
| *sRaw* (kPa/s^2^)^†^ | 0(-0.005:0.004) | 0.829 |  | -0.002(-0.007:0.004) | 0.547 |  | 0.002(-0.006:0.01) | 0.568 |
|  | **Exp (β Estimate) (95%CI)** | **P value** |  | **Exp (β Estimate) (95%CI)** | **P value** |  | **Exp (β Estimate) (95%CI)** | **P value** |
| *FeNO (ppb)*^‡^ | 0(-0.005:0.004) | 0.888 |  | 0(-0.005:0.006) | 0.944 |  | -0.002(-0.011:0.006) | 0.61 |
| *PD20 (µg)*^‡^ | 0.006(-0.005:0.017) | 0.288 |  | 0.01(-0.005:0.025) | 0.184 |  | 0.01(-0.029:0.048) | 0.621 |
| * Significant *P*-value ≤ 0.05  Key  FeNO = Fractional Exhaled Nitric Oxide, FEV1 = Forced Expiratory Volume 1 second, FVC = Forced Vital Capacity, HGS = Handgrip Strength, MMEF = Maximal Mid-expiratory Flow, PD20 = Provocation Dose of methacholine causing a drop of 20% in FEV1, sRaw = Specific Airway Resistance | | | | | | | | |

| Table e9. Association between handgrip strength lung function and allergy outcomes - unadjusted  Multiple linear regression was used for continuous outcomes^†^, multiple log-linear regression was used for log transformed continuous outcomes‡ and multiple logistic regression was for binary outcomes^§^. | | | | | | | | |
| --- | --- | --- | --- | --- | --- | --- | --- | --- |
|  | **All** |  |  | **Male** |  |  | **Female** |  |
| Outcome | **β estimate(95%CI)** | ***P* value** |  | **β estimate(95%CI)** | ***P* value** |  | **β estimate(95%CI)** | ***P* value** |
| *FEV1 (L)*^†^ | 0.05 (0.05-0.06) | <0.001* |  | 0.02 (0.01-0.04) | <0.001* |  | 0.03 (0.02-0.04) | <0.001* |
| *FVC (L)*^†^ | 0.06 (0.06-0.07) | <0.001* |  | 0.03 (0.01:0.04) | <0.001* |  | 0.04 (0.02-0.05) | <0.001* |
| *MMEF (L)*^†^ | 0.05 (0.04-0.06) | <0.001* |  | 0.03 (0.01-0.05) | 0.008* |  | 0.03 (0-0.05) | 0.032* |
| *FEV1/FVC*^†^ | 0.0 (0.0-0.0) | 0.092 |  | 0.0 (0.0-0.0) | 0.872 |  | 0.0 (0.0-0-0) | 0.513 |
| *sRaw* (kPa/s2)^†^ | 0.0 (0-0.01) | 0.142 |  | 0.0 (-0.01-0.0) | 0.471 |  | 0.0 (-0.01-0.01) | 0.572 |
|  | **Exp (β Estimate) (95%CI)** | **P value** |  | **Exp (β Estimate) (95%CI)** | **P value** |  | **Exp (β Estimate) (95%CI)** | **P value** |
| *FeNO (ppb)*^‡^ | 1.04 (1.02-1.06) | 0.001* |  | 1.00 (0.96-1.05) | 0.869 |  | 0.99 (0.93-1.05) | 0.747 |
| *PD20 (mcg)*^‡^ | 1.02 (1.01-1.04) | 0.009* |  | 1.02 (0.98-1.05) | 0.283 |  | 1.02 (0.97-1.06) | 0.454 |
| * Significant *P*-value ≤ 0.05  Key  FeNO = Fractional Exhaled Nitric Oxide, FEV1 = Forced Expiratory Volume 1 second, FVC = Forced Vital Capacity, HGS = Handgrip Strength, MMEF = Maximal Mid-expiratory Flow, PD20 = Provocation Dose of methacholine causing a drop of 20% in FEV1, sRaw = Specific Airway Resistance | | | | | | | | |

**Figure e1**: **ROC curves showing classification of asthma status dependent on FEV1 with versus without handgrip strength.**
Two different curves are shown in the same plot, one where FEV1 is adjusted for age, height, and weight and one where FEV1 is adjusted for age, height, weight, and handgrip strength. The P value stated in the plot is for the difference between areas under the curve for these two models.


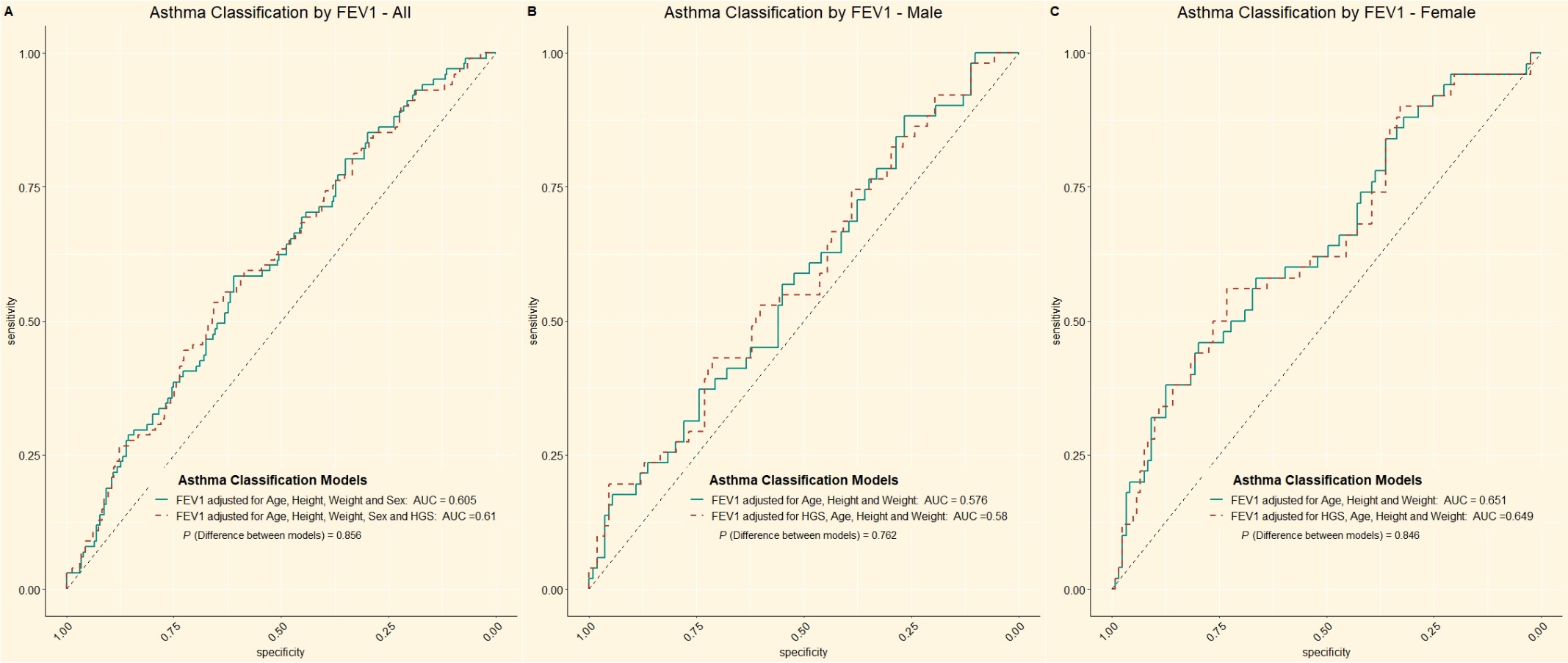


**Key**

**AUC** = Area under the curve, **FEV1** = Forced Expiratory Volume 1 second, **HGS** = Handgrip Strength.

**Figure e2. Heatmaps of Handgrip Strength Measures for Males and Females.**

Heatmap based on Pearson correlation. Correlations are expressed as correlation coefficient R. The red colour reflects a positive correlation. The darker the red colour is, the greater the correlation coefficient.


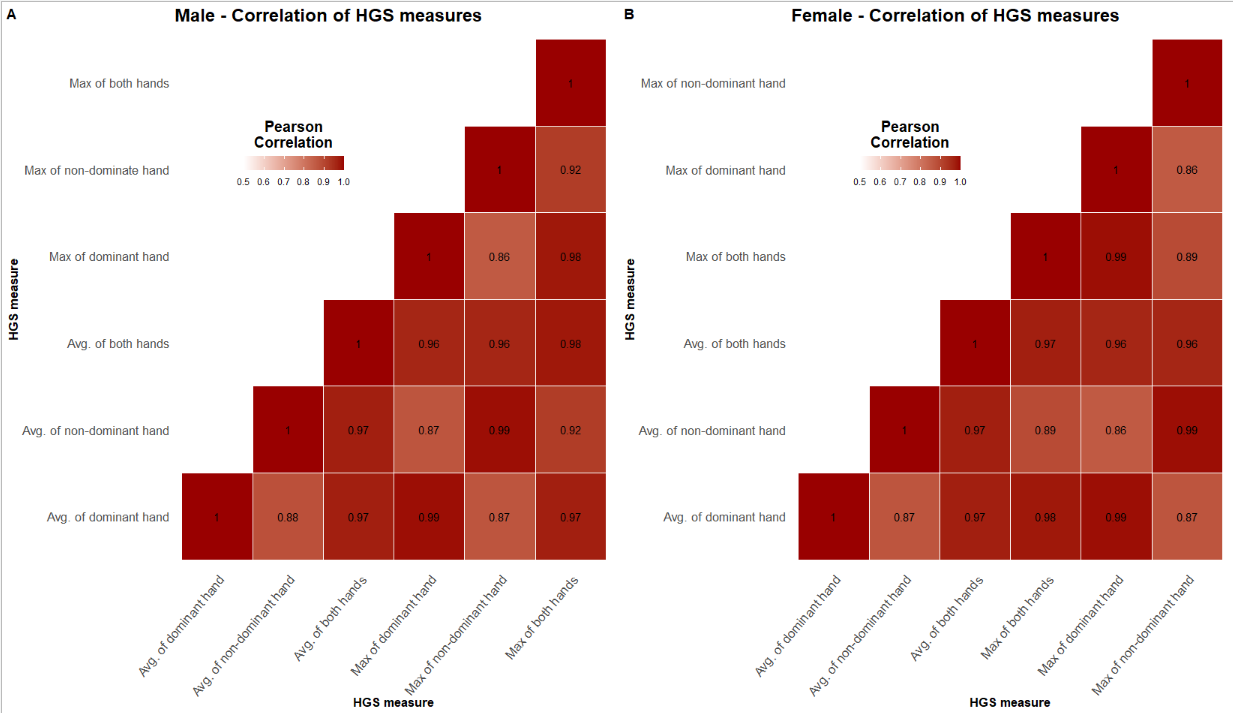


**Key**

**HGS** = Handgrip strength, **Max of both hand** = The maximal measurement of the 6 measurements from both hands, **Max of non – dominant hand** = Maximum HGS values of 3 measurements on the non-dominant hand. **Max of the dominant hand** = Maximum HGS values of 3 measurements on the non-dominant hand, **Avg. both hands** = The mean average of the 6 measurements from both hands**, Avg. of non – dominant hand** = The mean average of HGS values of 3 measurements on the non-dominant hand, **Avg. of the dominant hand** = The mean average of HGS values of 3 measurements on the non-dominant hand.
